# Clinimetric properties of the Chinese version of the subjective incompetence scale: an extended paradigm

**DOI:** 10.3389/fpsyt.2026.1825824

**Published:** 2026-10-29

**Authors:** Boheng Zhu, Yanping Duan, John M. de Figueiredo, Danilo Carrozzino, Jiaojiao Hu, Tao Li, Jing Wei

**Affiliations:** 1 Department of Psychological Medicine, Peking Union Medical College Hospital, Chinese Academy of Medical Sciences & Peking Union Medical College, Beijing, China; 2 Department of Psychiatry, Yale University School of Medicine, New Haven, CT, United States; 3 Department of Psychology “Renzo Canestrari”, Alma Mater Studiorum University of Bologna, Bologna, Italy

**Keywords:** clinimetrics, demoralization, psychological distress, relative validity, subjective incompetence

## Abstract

**Objectives:**

This is the first validation study to evaluate the measurement properties (i.e., construct, concurrent, incremental, and relative validity) and determine the clinical utility of the Chinese version of the 12-item subjective incompetence scale (SIS-12) based on the science of clinimetrics.

**Methods:**

Consecutive general hospital inpatients (n= 232) in the Departments of Neurology and Obstetrics & Gynecology were assessed for SIS-12, along with measures of anxiety, depression, stress levels, and demographic information. Mokken analysis was employed to evaluate construct validity, while relative validity was assessed through relative weight analysis.

**Results:**

Mokken analysis indicated inadequate construct validity for items 1, 4, 8, and 10, leading to the development of an 8-item version of the scale (SIS-8), which was found to have optimal scalability. The SIS-8 also entailed the clinimetric properties of concurrent validity, incremental validity, and relative validity.

**Conclusion:**

The findings of the present clinimetric validation indicate that the SIS-8 can potentially be used both as a screening measure for the early detection of individuals at increased risk for demoralization and as a dimensional measure to assess the severity of feelings of subjective incompetence. This dual utility suggests its relevance in both clinical research and practice, particularly in managing inpatients suffering from psychological distress.

## Background

Sense of competence/efficacy has gained considerable recognition as a critical factor in healthcare practice, given its well-documented contribution to multiple health behaviors and outcomes across diverse cultures (1, 2). Evidence from different patient populations-spanning both outpatient and inpatient settings- consistently suggests that a stronger sense of competence/efficacy correlates with improved treatment adherence, encompassing not only medication compliance (3, 4) but also beneficial changes in lifestyle, such as increased exercise (5), better dietary habits (6), and decreased smoking and drinking (7). Moreover, a heightened sense of competence/efficacy is predictive of a range of positive physical health outcomes, such as enhanced glycemic control in patients with diabetes (3, 8), and reduced serum phosphorus levels in patients undergoing chronic hemodialysis (9), as well as improvements in mental health conditions, including decreased depressive symptoms (10), elevated quality of life (11, 12), and greater confidence in healthcare providers and social support (13, 14; 16).

Recently, Ponton (15) argued that a weak sense of efficacy should not be equated with a strong sense of inefficacy, as the former is rooted in insufficient efficacy-enhancing information, while the latter arises from an abundance of efficacy-diminishing information. Similarly, an empirical investigation on burnout among university students underscored the distinction between low efficacy and inefficacy (17). However, the current clinical assessment methods overlook this distinction, often employing a reversed competence/efficacy scale to measure the sense of incompetence or inefficacy (17, 18), highlighting the need to develop valid instruments for accurately assessing incompetence and inefficacy.

The concept of subjective incompetence (SI) has been defined for the first time by de Figueiredo and Frank (19) as a state experienced by individuals who perceive themselves as unable to perform the tasks and express the feelings they consider appropriate in a stressful situation. In clinical psychosomatics, SI has been recognized as the clinical hallmark of demoralization, a prolonged psychological state marked by distressing feelings often expressed in terms such as “it is too much for me”, “I cannot take it anymore”, ascribed by the individual to failures or deficiencies in one’s environment (helplessness) and/or to one’s own personal inadequacies for which nothing can be done (hopelessness) (20, 21). SI represents the initial stage of the demoralization cascade, which may progress to evoke feelings of helplessness and hopelessness successively, potentially culminating in suicidality (de 22, 23). In contrast to the constructs of perceived competence and self-efficacy, SI does not sharply distinguish between individuals and their behaviors (i.e., it is not conceptualized as a proximate cognition to a target behavior) (24), thereby not being confined to specific behaviors or domains (e.g., diabetes self-management, dieting, exercise, and etc.) (19).

Cockram et al. (25, 26 developed a 12-item self-assessment scale for subjective incompetence (SIS-12) and evaluated its psychometric properties among cancer patients in the United States. The scale demonstrated good internal consistency and a strong association between SI and maladaptive coping strategies, including self-blame and behavioral disengagement. Subsequent research using the SIS-12 indicated that it can significantly predict treatment outcomes in individuals with eating disorders in Italy (27). Moreover, among U.S. patients with Parkinson disease, SI was found to be a crucial factor contributing to demoralization, independent of depression and anxiety (23). Although SI is generally considered a unidimensional construct, the factorial structure of the SIS-12 was not explicitly examined in these studies. However, a recent study by Murri et al. (28) in the Italian general population supported a three-factor structure for the SIS-12 based on exploratory factor analysis: subjective incompetence, inability to plan, and inability to deal. Despite these findings and promising clinical implications, there remains a lack of validated Chinese tools for assessing SI.

The term “clinimetrics”, which results from the combination of the words “clini” (that stands for clinical) and “metrics” (that refers to a measurement process based on specific criteria), was originally coined by Alvan R. Feinstein (29, 30) to introduce the science of clinical measurements (31, 32). This innovative clinically-based approach emphasizes the clinical utility of indices and rating scales, particularly when used to evaluate a wide range of clinical issues, for example, symptom severity, sensitivity to changes in clinical (i.e., psychotherapy or drug) trials, differentiation between active therapy and placebo, as well as distinction between different patient groups, detecting clinically relevant changes in drug or psychotherapy trials, and many other clinical factors that demarcate major prognostic and therapeutic differences (31, 33, 34). Recently, a panel of international experts has proposed a set of clinimetric criteria that apply to the development of new and to the validation of existing patient-reported outcome measures (34). In particular, the authors of this consensus paper placed scalability at the core of construct validity, defining it as the evidence that each item included a clinimetric index or rating scale provides distinctive or unique clinical information and belongs to the same underlying clinical dimension (34). In the same vein, internal consistency, which measures the degree to which items in a rating scale are correlated, is not necessarily required within the clinimetric criteria (34). Scalability is conventionally tested using Mokken analysis to determine whether individual items and the total score of the assessment instrument provide a statistically sufficient and clinically valid measure of the severity of the latent clinical dimension under examination (34, 35).

Following this clinimetric principle, we intend to extend the current clinimetric criteria for validity by introducing “relative validity”. The term is defined as the ability of a particular instrument to hold importance for clinical decisions (i.e. the extent to which the measure contributes to the variables of interest) when taking the other relevant measures into consideration. Relative validity can be seen as an extension of incremental validity, as the latter establishes if a measure adds unique information, whereas the former determines how clinically important the information is, in relation to other existing assessments. Relative validity may have a significant bearing on clinical practice. In busy clinical settings and the upcoming era of process-based psychotherapy (36), relative validity may enable us to carefully select the most impactful measures while keeping them to a minimum. Relative validity can be quantified by relative importance statistics in multiple regression analyses, such as Johnson’s relative weights (37) and dominance analysis (38). Moreover, the magnitude of relative validity can be qualitatively determined by applying Cohen’s criteria for effect size (39).

Based on this conceptual framework, the present study endeavors to validate the Chinese version of SIS-12 through the utilization of an extended clinimetric paradigm among general hospital inpatients in Beijing, China. To this end, the current version of the SIS-12 was assessed for a set of clinimetric properties including construct, concurrent, incremental, and relative validity, among inpatients from Neurology and Obstetrics & Gynecology departments of a general hospital in China. These departments were specifically selected based on previous research indicating a high prevalence of demoralization in those patient populations within the Chinese healthcare context (40, 41). This approach allows for a comprehensive and rigorous assessment of the scale’s applicability not only in the Chinese clinical context but also in all those studies and clinical settings where the improved detection of subjective incompetence may determine major prognostic and therapeutic outcomes.

## Methods

### Study design and sample size determination

This is a cross-sectional study conducted from July 2022 to February 2023. Sample size was estimated based on methodological recommendations (42) for sample size determination in validation studies of patient-reported outcome measures, which suggested a sample size of at least 100 subjects.

### Participants

Participants were consecutive inpatients recruited at the Neurology and Obstetrics & Gynecology Departments of a general hospital in Beijing, China. Inclusion criteria were a) patients admitted to the Neurology and Obstetrics & Gynecology Departments of the hospital during the study period, b) aged 14 or over, and c) willing to participate. Exclusion criteria include a) patients with a hospital stay of fewer than 24 hours, and b) patients who were unable to complete the relevant assessments due to language, writing, or reading barriers. All participants provided written informed consent, ensuring their full understanding of the study’s purpose and potential risks. No incentives were offered for participation in the study. The study was conducted according to the World Medical Association Declaration of Helsinki and approved by the Ethical Committee of the participating hospital.

### Data sources and assessments

Trained research assistants administered the questionnaires electronically via smartphone immediately following clinic appointments. The program required responses to each question before allowing participants to proceed. Participants provided information on their age, sex, race/ethnicity, marital status, education level, cigarette smoking habits, and alcohol consumption patterns.

SIS-12 is a 12-item self-assessment tool designed to evaluate feelings of SI (25). For each item, the response is provided on a 4-point Likert scale ranging from 0 (none of the time) to 3 (most or all of the time), reflecting the frequency of the corresponding experience (e.g. “puzzled, indecisive, and uncertain as to what actions to take”). The sum score of the 12 items (with items 1, 4, and 10 being reverse-scored) is calculated, yielding a range from 0 to 36, with higher scores indicating greater levels of SI (25). For this study, a proficient bilingual native Chinese speaker undertook the translation of the original English version into Chinese. Subsequently, two senior psychiatrists scrutinized and revised the translation to guarantee the naturalness and cultural appropriateness of each item in the Chinese context. Following this, two bilinguals, unaware of the original English version, performed a back-translation from Chinese to English. Then, the senior author of the original version of the SIS-12 meticulously reviewed and provided feedback on the back-translation, ensuring that the meaning of each item aligns with that of the original scale.

The Patient Health Questionnaire-9 (PHQ-9) (43, 44), a widely employed self-report scale comprising 9 items, was used to assess depression, on a scale ranging from 0 (not at all) to 3 (nearly every day). The total score, calculated by summing all item scores, reflects depression severity (higher scores indicate greater severity).

Anxiety was evaluated using the subscale from the Union Physio-Psycho-Social Assessment Questionnaire (UPPSAQ70) (45; Wang et al., 2024). This self-report questionnaire was specifically designed to measure overall health in the psychological, physical, and social domain. It has undergone validation within the general hospital context of China (45; 46). The subscale comprises eight items aimed at quantifying the severity of anxiety (e.g., “I feel jittery, worried, or frightened”). Each item is evaluated on a 4-point Likert scale from 0 (not at all) to 3 (most or all of the time), resulting in a total score ranging from 0 to 24. A higher score indicates a greater severity of anxiety.

Stress levels were measured using the Life Events Scale (LES) (47), a 48-item self-assessment scale designed to gauge the cumulative impact of life events across three domains (i.e., family life, work/study, and social/other). For each event, stress scores were computed by multiplying three factors: (1) impact severity, (2) duration of impact, and (3) frequency of occurrence. The total stress score was derived by summing all item scores, with higher scores reflecting greater stress levels.

Suicidal ideation was assessed using item 9 of the PHQ-9 (43, 44), employing a 4-point response scale ranging from 0 (not at all) to 3 (nearly every day).

### Statistical analyses

Descriptive statistics (mean, median, and frequency) were used to describe the sample characteristics, as appropriate. We used the Shapiro-Wilk test to check for normal distribution of the continuous variables. Clinimetric properties of the SIS-12 were assessed using the following statistical analyses:

#### Construct validity

Mokken analysis was employed to assess the construct validity of the SIS based on the double monotonicity model (DMM) (48). This involved a comprehensive examination of scalability coefficients, as well as local independence, monotonicity, and Invariant Item Ordering (IIO) assumptions. The analytical process consisted of the following steps: 1) Scalability coefficients were evaluated at both the item (Hj) and scale levels (H) using a predetermined cutoff of 0.30. As suggested by Mokken (49), an item or a scale is deemed unscalable if either the scalability coefficient at the item level (Hj) or the scale level (H) is less than 0.3. If the overall scalability of the scale fell below 0.3, the automated item selection procedure (AISP) method was utilized to generate a refined scale, with MINVI sizes (i.e., lower bound of scalability coefficient) of 0.3, 0.4, and 0.5; 2) Monotonicity and IIO assumptions were examined using statistical significance and the *crit* index for each item. A significant violation was defined as having either the number of statistically significant violations *(#zsig or #tsig)* ≥1 or the *crit* value ≥ 40, as proposed by Stochl et al. (50). Items that violated the monotonicity and IIO assumptions were removed from the scale one at a time, starting with the item having the highest *crit* value and continuing until no further violations were found; 3) local independence was tested through conditional association procedure with W1, W2, and W3 statistics. Pairs of items with local correlation were marked by the procedure and subsequently eliminated one by one until no violating pairs were detected; 4) the scalability coefficient for the final version of the scale was computed, when applicable. In addition, we conducted a confirmatory factor analysis (CFA) on the final version of the scale to determine whether the results aligned with those from the Mokken analysis. CFA applied MLM estimation with scaled fit indices to account for non-normality (Shapiro-Wilk p < 0.05).

#### Concurrent validity

Spearman correlation coefficients were employed to evaluate the relationship between the SIS-12 and measures of depression, anxiety, suicidal ideation, and stress. Moderate correlations (r= 0.4-0.6) were anticipated for the association of SI with depression and anxiety, while small to moderate correlations (r= 0.2-0.4) were expected for its relationship with suicidal ideation and stress (23).

#### Incremental validity

Hierarchical regression analysis assessed whether subjective incompetence contributed to depression and anxiety, above the variance explained by LES. Incremental validity was estimated by the increase of R^2^ (ΔR^2^) following the inclusion of SI to LES.

#### Relative validity

A method originated from Johnson’s relative weights (37) was used to assess the relative importance of SIS, together with stress, on depression and anxiety. The magnitude of the validity was determined in accordance with Cohen’s criteria (i.e. R^2^<0.02, very weak; 0.02≤R^2^<0.13, weak; 0.13≤R^2^<0.26, moderate; R^2^≥0.26, large) (39).

Mokken analysis, relative weight analysis, and CFA were conducted using the “mokken” (51), “rwa” (52), and “lavaan” (53) packages in R Studio, respectively; other statistical analyses were performed using SPSS version 23. The statistical significance level was set at p ≤ 0.05, and all statistical tests were two-tailed.

## Results

### Sample characteristics

The final sample consisted of a total of 232 participants (response rate = 96.7%) with no missing data. The majority were female (74.6%), hospitalized in the neurology ward (50.0%), married (79.7%), and with a high school degree or higher (78.9%). The age range of the sample was 14–82 years (mean=42.09 years, SD = 13.77 years). Only 11 subjects (4.7%) were cigarette smokers; 94 (40.5%) reported drinking alcoholic beverages; and 67 (28.9%) received psychological medication or psychotherapy within the last 6 months. The Shapiro-Wilk test indicated non-normal distributions for LES, SIS-12, PHQ-9, and UPPSAQ-70 anxiety scores (p< 0.05). The median SIS-12 score was 12 (IQR = 9–15; see **Table 1**).

**Table 1:** Sample characteristics.

	N=232	
Categorical variables		
	N	%
Gender (Male)	59	25.4
Department (neurology)	116	50.0
Health insurance (yes)	218	94.0
Marital state (married)	185	79.7
Education level (High school or higher)	183	78.9
Family income		
<4000 yuan	22	9.5
4000–8000 yuan	74	31.9
>8000 yuan	136	58.6
Currently employed	125	53.9
Living alone	23	9.9
Current cigarette smoking	11	4.7
Current alcohol drinking	94	40.5
PMP administration within the last 6 months	67	28.9
Suicidal ideation		
Not at all	178	76.7
Several days	51	22.0
More than half the days	3	1.3
Nearly every day	0	0
Continuous Variables		
	MEAN	SD
Age	42.09	13.77
	MEDIAN	IQR
LES	13	0-48
UPPSAQ-70 anxiety	5	3-8
PHQ-9	5	2-7
SIS-12	12.00	9-15

PMP, Psychotropic medication and psychotherapy; LES, Life Events Scale; UPPSAQ-70, Union Physio-Psycho-Social Assessment Questionnaire-70; PHQ-9, Patient Health Questionnaire-9; SIS-12, Subjective Incompetence Scale-12.

### Construct validity

**Table 2** shows the scalability coefficients of the SIS-12. The overall scale coefficient indicates a poor level of scalability for the 12-item scale (H = 0.26). Individual item scalability (Hi) for items 1, 2, 4, 7, and 10, were below the cut-off of 0.30, indicating they were unscalable with the other items. Further automatic item selection procedures with different MINVI sizes (0.3-0.5) consistently yielded a 9-item scale, where items 1, 4, and 10 were omitted because of weak scalability with the other items. No significant violations of monotonicity and IIO were detected (see **Table 3**) for the 9-item scale, with a *crit* value of zero and no statistically significant violations for each item. Conditional association procedure marked significant local dependence between item pairs 2 and 8, 5 and 7, as well as between items 5 and 8 according to W3 statistics (see **Supplementary Table S1**). Item 8 was removed from the scale by the procedure and the re-analysis of the 8-item scale detected no local dependence for each pair of items (see **Supplementary Table S2**). Finally, **Table 4** displays the scalability coefficients of the 8-item scale (SIS-8). The 8-item scale shows adequate scalability in overall (H = 0.56) and for each item (Hi range=0.47 to 0.62). Additionally, the one-factor model of SIS-8 demonstrated acceptable fit in confirmatory factor analysis (scaled CFI = 0.929, scaled TLI = 0.901, scaled RMSEA = 0.076; see **Supplementary Table S3**).

**Table 2:** Initial scalability and identification of Mokken scales for SIS using the aisp function.

Items	MINVI size^a^			H*i*(se)
	0.3	0.4	0.5	
1	2	2	2	**0.12(0.05)**
2	1	1	1	**0.28 (0.04)**
3	1	1	1	0.37 (0.04)
4	2	2	2	**0.00(0.06)**
5	1	1	1	0.36 (0.04)
6	1	1	1	0.38 (0.04)
7	1	1	1	**0.27 (0.05)**
8	1	1	1	0.31 (0.05)
9	1	1	1	0.36 (0.04)
10	2	2	2	**0.06(0.05)**
11	1	1	1	0.41 (0.03)
12	1	1	1	0.38 (0.04)
Scale H: 0.26(0.03)				

Bold indicates H*i* < 0.3; se, standard error; ^a^ the numbers in each column indicate the scale to which the item belongs according to different levels of MINVI size.

**Table 3:** Output of assessment of monocity and IIO for 9-item SIS.

Items	Monocity coefficients				IIO coefficients			
	#ac	#vi	crit	#zsig	#ac	#vi	crit	#tsig
2	2	0	0	0	8	0	0	0
3	3	0	0	0	8	0	0	0
5	3	0	0	0	8	0	0	0
6	2	0	0	0	8	0	0	0
7	3	0	0	0	8	0	0	0
8	3	0	0	0	8	0	0	0
9	2	0	0	0	8	0	0	0
11	1	0	0	0	8	0	0	0
12	3	0	0	0	8	0	0	0

#ac, number of active pairs that were investigated; #vi, number of violations in which the item is involved; crit, Crit value; #zsig/#tsig, number of statistically significant violations.

**Table 4:** Scalability coefficients for the revised 8-item version of the SIS.

Items	Coefficients	
	H*i*	se
2	0.47	0.06
3	0.60	0.05
5	0.55	0.05
6	0.57	0.05
7	0.52	0.06
9	0.54	0.05
11	0.62	0.04
12	0.59	0.05

Scale H: 0.56(0.04).

se, standard error.

### Concurrent validity

**Figure 1** presents the concurrent validity analysis of the revised 8-item version of SIS. As hypothesized, scores of the SIS-8 (median=7, IQR = 3-8) show significant correlations with measures of depression and anxiety, exhibiting moderate correlations (r= 0.4-0.6). Additionally, the scores also demonstrate small to moderate correlations (r= 0.2-0.4) with suicidal ideation and life events, confirming our expectations.

**Figure 1:**
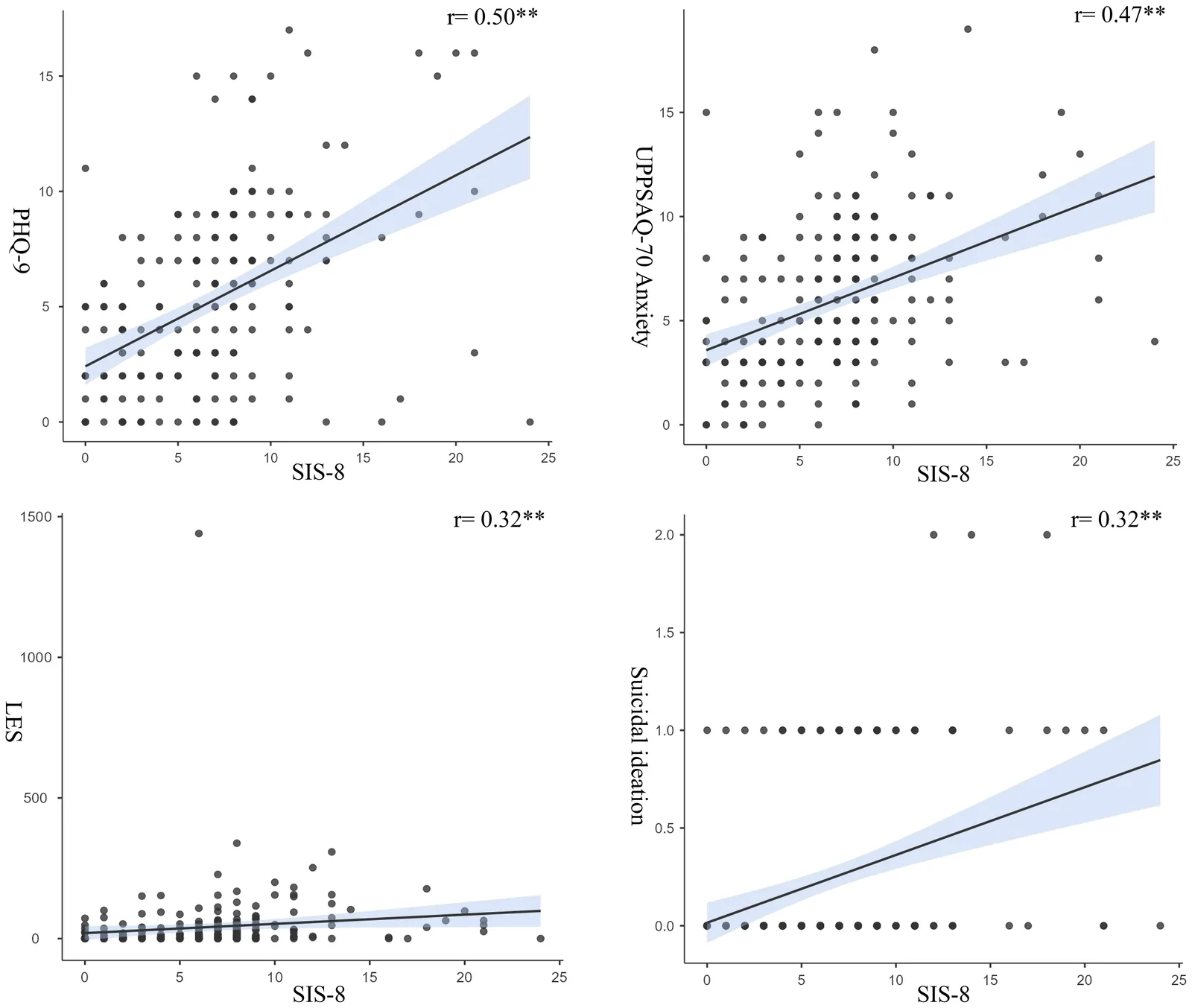
Concurrent validity of SIS-8 using Spearman’s rho correlation coefficients. PHQ-9, Patient Health Questionnaire-9; GAD-7, General Anxiety Disorder Scale-7; SIS-8, Subjective Incompetence Scale-8; UPPSAQ-70, Union Physio-Psycho-Social Assessment Questionnaire-70; LES, Life Events Scale; **p-value < 0.001.

### Incremental and relative validity

As illustrated in **Table 5**, the SIS-8 shows significant incremental validity over stress to predict depression (Δr^2^ = 0.19) as well as anxiety symptoms (Δr^2^ = 0.16). Moreover, the SIS-8 demonstrated moderate relative validity to count for anxiety (r^2^ = 0.14) and depression (r^2^ = 0.21), together with stress measured by LES.

**Table 5:** Incremental and relative validity of SIS-8.

	Coefficients				
Predictors	B	se	p	R^2^	p
Outcome: Anxiety (UPPSAQ-70)				**0.22**	<.001
LES	0.01	0.00	0.001	**0.05^a^**	
SIS-8	0.33	0.05	<0.001	**0.17 ^a^**	
ΔR^2^	**0.16**				
Outcome: Depression (PHQ-9)				**0.26**	<.001
LES	0.01	0.00	0.001	**0.05 ^a^**	
SIS-8	0.39	0.05	<0.001	**0.21 ^a^**	
ΔR^2^	**0.19**				

Statistically significant values (p < 0.05) are in bold. ^a^Indicates the Johnson’s relative weights.

## Discussion

This is the first validation study in which the clinimetric properties of the Chinese version of the SIS-12 have been assessed using an extended paradigm. The measurement property of “relative validity” was introduced and its unique implications for the validation of clinical measures were discussed. The study’s strengths thus include the application of innovative clinimetric criteria and robust statistical methods, particularly comprehensive Mokken analysis (48) and relative weight analysis (37), to determine the clinical utility of the SIS-12.

The analyses revealed that the total score of a short version of the scale, the SIS-8, which only included 8 items, had optimal scalability, thus being a highly valid or unidimensional measure of subjective incompetence. In other words, findings of the present clinimetric validation support the use of the SIS-8 not only as a screening measure for early detection of individuals at increased risk for development of demoralization but also as a dimensional measure to assess the severity of feelings of subjective incompetence. The use of the SIS-8 has the potential to help clinical investigators and clinicians differentiate healthy or homeostatic reactions to adverse life events from experiences and manifestations of subjective incompetence that may require clinical attention.

In terms of construct validity, items 1 (i.e., “Were you able to plan and initiate concerted action as well as you thought you could?”), 4 (i.e., “Did you feel that you could continue to plan, strategize and initiate appropriate action?”), and 10 (i.e., “When you were experiencing this situation, did you feel that you could continue to carry out your usual activities [such as doing your housework, concentrating or visiting people]?”) of the original version of the SIS-12 had poor scalability, thus indicating that these items did not belong to the underlying dimension of subjective incompetence that they intended to measure. Examining the item content suggests that these items may reflect an underlying construct of “perceived inability to react” (i.e., being unable to plan, take action, and engage in daily activities) rather than pure feelings of subjective incompetence. This is consistent with the evidence that individuals experiencing demoralization usually maintain the capacity to react (20, 21). As Fava and his research group (20, 21) observed, depressed patients experience a lack of motivation and drive, while in a demoralized person they are usually intact, even though they may be fluctuating and inhibited by helplessness. Similarly, de Figueiredo et al. (23) argued that the magnitude of motivation (i.e., willingness, desire, or interest) is preserved in demoralization, though the direction of motivation (i.e., course of action) is thwarted by uncertainty about how to get out of the predicament. It is noteworthy that the latter does not imply a loss of ability to plan and act in a general manner, but rather a perception that there is a narrowing of pathway toward the desired outcomes. This interpretation is well exemplified by the case that people who are in a hopeless state (i.e., an advanced stage of demoralization) can typically plan and even commit suicide (54). Our findings are also consistent with previous research viewing subjective incompetence as a one-dimensional construct (19, 25). In the present study, after the exclusion of the three items reflecting the perceived inability to react, the SIS-8 was found to be a unidimensional index of subjective incompetence.

Another plausible reason for the three items displaying inadequate scalability coefficients could be their reverse content in contrast to the other items, potentially causing reverse item bias. While utilizing reverse-worded items in assessments can have a number of advantages (e.g., controlling acquiescence and providing wider coverage of a construct’s content), it can also lead to problematic responses given acquiescence, careless responding, and confirmation bias (55, 56). This implies that, in order to calibrate the bias, these three items may benefit from adjustment to non-reverse content for subsequent validation (57). In line with this methodological recommendation, Bech (58), in his editorial on clinimetric dilemmas in outcome scales for mental disorders, suggested avoiding the use of items worded in oppositive directions that have to be reversed in the scoring process. Regarding item 8 (i.e., “Did you change your mind about your ability to deal with similar situations?”), it exhibited significant local dependence with items 2 (i.e., “Were you puzzled, indecisive and uncertain as to what actions, if any, you should take?”) and 5 (i.e., “Did you feel that you were running out of ideas to handle the situation?”), leading to its exclusion through the stepwise automated procedure. This suggests that item 8 may benefit from a rewording not only to avoid clinical redundancy between items but also to allow each item of the scale to express its particular piece of clinical information (59).

As to the concurrent validity, the findings of the present study confirmed our hypotheses, indicating the differential role of subjective incompetence in depression, anxiety, suicidality, and stress. This aligns with the conceptualization of subjective incompetence as an early sign of demoralization (22, 23). Moreover, consistent with our research expectations, the findings from the incremental and relative validity highlighted the clinical utility of SIS-8, suggesting that this index may provide a unique and clinically significant contribution to psychological distress outcomes. Specifically, SIS-8 emerges as a valuable instrument for informing the care of hospitalized patients who present symptoms of depression, anxiety, and suicidality. This finding corresponds to a previous study by Tecuta et al. (27) on patients with eating disorders, where SI demonstrated an independent predictive effect on changes in eating disorder symptomatology.

The study has several limitations that warrant attention. First, it is crucial to acknowledge the high heterogeneity among the inpatient population within the comprehensive hospital setting. Consequently, the external validity of the study’s conclusions should be interpreted with caution. Second, the lack of self-efficacy assessment in this study prevents our ability to disentangle the unique contributions of subjective incompetence from a weak sense of efficacy. Third, the present study did not assess patients’ disease-related characteristics— including disease duration, severity, and psychotropic use—, which may influence subjective incompetence and thus confound the validity of the SIS. Fourth, the absence of qualitative assessment for demoralization rules out the possibility of establishing cut-off values for the scale, thereby restricting its clinical applicability and guidance (20). Future investigations are warranted to fill this gap and clarify the relationship between subjective incompetence and demoralization. Finally, the cross-sectional design of the study precludes the investigation of predictive validity and clinimetric sensitivity of the scale. This limitation underscores the necessity for future refinements, possibly through longitudinal approaches, to enhance our understanding of the scale’s performance over time.

## Conclusions

The findings of this clinimetric validation indicate that the revised 8-item Chinese version of the Subjective Incompetence Scale (SIS), the SIS-8, is a valid measure that can be used in clinical research and practice to assess the degree of feelings of subjective incompetence. This easy-to-use and no-time consuming clinimetric index holds promise as a valuable tool in the management of inpatients experiencing psychological distress. It may serve both as a screening measure to identify individuals at heightened risk of demoralization and as a dimensional tool to distinguish between healthy adaptive responses to adversity- such as the burden imposed by the medical condition and associated life disruptions from clinically significant subjective incompetence requiring intervention.

Nonetheless, the findings of this study should be cautiously considered due to factors such as the heterogeneity of the hospitalized patient population, challenges in establishing cut-off values for the scale, and the cross-sectional study design. Further research is needed to refine the scale’s clinical utility and verify its robustness in diverse clinical contexts.

## Data Availability

The raw data supporting the conclusions of this article will be made available by the authors, without undue reservation.
