# Supplementary material for: Clinimetric properties of the Chinese version of the subjective incompetence scale: an extended paradigm
Source: Front Psychiatry. 2026 Oct 29;17:1825824. doi: 10.3389/fpsyt.2026.1825824 (PMC13754913; doi:10.3389/fpsyt.2026.1825824)
Supplement: Supplementary file 1 [file Table1.docx]

**The Subjective Incompetence Scale**

Instructions: Below are several statements about how people may feel or behave when they try to deal with a stressful situation they are facing. Please read each statement carefully and choose the numbered response that best describes how you felt or behaved that way during the past week including today. For example, if you experienced the feelings described none of the time, you would indicate so by choosing 0 (zero); if you experienced the feelings described most or all of the time, you would indicate so by choosing 3 (three).

0=None of the time, 1=A little bit of the time, 2=A good bit of the time, 3=Most or all of the time.

1. Were you able to plan and initiate concerted action as well as you thought you could?
2. Were you puzzled, indecisive and uncertain as to what actions, if any, you should take?
3. Did you feel you were facing a quandary, a dilemma or a predicament?
4. Did you feel that you could continue to plan, strategize and initiate appropriate action?
5. Did you feel that you were running out of ideas to handle the situation?
6. Did you reach a point where you became convinced that the situation was out of your hands?
7. Did you reach a point where you became convinced that someone else would be able to handle the situation better than you would?
8. Did you change your mind about your ability to deal with similar situations?
9. Did the situation convince you that your assumptions about other people, such as their eagerness to help you, were no longer certain?
10. When you were experiencing this situation, did you feel that you could continue to carry out your usual activities (such as doing your housework, concentrating or visiting people)?
11. While you were dealing with the situation, did the situation shake your confidence in your ability to deal with future problems you may encounter in your life?
12. Has the situation left you with new doubts about your ability to deal with any future problems you may encounter in your life?

*Source.* Cockram, C. A., Doros, G., & de Figueiredo, J. M. (2009). Diagnosis and measurement of subjective incompetence: The clinical hallmark of demoralization. Psychotherapy and Psychosomatics, 78(6), 342–345. [https://doi.org/10.1159/000235737](https://psycnet.apa.org/doi/10.1159/000235737)

The Chinese version of subjective incompetence scale(12 items)：

主观无能感量表(12条目)

以下是一些人们在试图处理他们所面临的压力情境时可能会有的感觉或行为的陈述。请仔细阅读每条陈述，选择最能描述你在过去一周（包括今天）的感觉或行为的数字回答。例如，如果你没有经历过所描述的感觉，你可以选择0；如果你大部分或全部经历过所描述的感觉，你可以选择3。

0-从来没有；1-偶尔；2-经常；3-大多数时候或总是

1. 你是否能够计划并采取相应的行动, 并如你认为你能的那样好？
2. 对于你应该采取何种行动，你是否感到困惑、优柔寡断和不确定？
3. 你是否认为自己正面临某种困境或窘境？
4. 你是否觉得你可以继续计划, 策划和发起适当的行动？
5. 你是否觉得处理这种情况，你已经无计可施?
6. 你是否达到了这样的程度，即你确信这种情况已经超出了你的能力范围？
7. 你是否达到了这样的程度，即你确信别人比自己能更好处理这种情况？
8. 你是否改变了关于自己处理类似情况能力的看法？
9. 这种情况是否让你相信你对他人的假设是真的？例如怀疑他们帮助你的热忱？
10. 当你遇到这种情况，你是否觉得你可以继续你的常规活动（例如：做家务、集中注意力或拜访他人）？
11. 当你在处理这种情况时，它是否动摇了你对自己未来处理可能出现的问题的信心？
12. 这种情况是否让你对自己处理未来生活中可能遇到的任何问题的能力产生了新的怀疑？

The adaptive Chinese version of subjective incompetence scale(8 items)：

修订版主观无能感量表(8条目)

以下是一些人们在试图处理他们所面临的压力情境时可能会有的感觉或行为的陈述。请仔细阅读每条陈述，选择最能描述你在过去一周（包括今天）的感觉或行为的数字回答。例如，如果你没有经历过所描述的感觉，你可以选择0；如果你大部分或全部经历过所描述的感觉，你可以选择3。

0-从来没有；1-偶尔；2-经常；3-大多数时候或总是

1.对于你应该采取何种行动，你是否感到困惑、优柔寡断和不确定？

2.你是否认为自己正面临某种困境或窘境？

3. 你是否觉得处理这种情况，你已经无计可施?
4. 你是否达到了这样的程度，即你确信这种情况已经超出了你的能力范围？
5. 你是否达到了这样的程度，即你确信别人比自己能更好处理这种情况？
6. 这种情况是否让你相信你对他人的假设是真的？例如怀疑他们帮助你的热忱？
7. 当你在处理这种情况时，它是否动摇了你对自己未来处理可能出现的问题的信心？
8. 这种情况是否让你对自己处理未来生活中可能遇到的任何问题的能力产生了新的怀疑？
