# Supplementary material for: Clinimetric properties of the Chinese version of the subjective incompetence scale: an extended paradigm
Source: Front Psychiatry. 2026 Oct 29;17:1825824. doi: 10.3389/fpsyt.2026.1825824 (PMC13754913; doi:10.3389/fpsyt.2026.1825824)
Supplement: Supplementary file 2 [file Table2.docx]

**Table S1. Local independence analysis of the 9-item subjective incompetence scale.**

| Index | Item | | | | | Item | | | | |
| --- | --- | --- | --- | --- | --- | --- | --- | --- | --- | --- |
|  |  | 2 | 3 | 5 | 6 | 7 | 8 | 9 | 11 | 12 |
| W1 | 2 |  | 7.77 | 5.03 | 5.28 | 4.73 | 1.16 | 3.33 | 5.66 | 4.68 |
|  | 3 | 0.77 |  | 3.50 | 5.40 | 0.60 | 1.00 | 3.75 | 5.10 | 2.70 |
|  | 5 | 2.23 | 3.41 |  | 4.79 | 1.72 | 0.53 | 3.11 | 5.51 | 2.68 |
|  | 6 | 0.59 | 1.92 | 1.33 |  | 1.09 | 0.97 | 0.50 | 1.81 | 1.54 |
|  | 7 | 1.70 | 2.92 | 2.04 | 5.16 |  | 2.12 | 3.07 | 5.36 | 2.55 |
|  | 8 | 1.48 | 3.54 | 2.67 | 6.39 | 3.07 |  | 4.77 | 7.60 | 3.47 |
|  | 9 | 1.09 | 2.97 | 1.88 | 4.92 | 1.45 | 1.55 |  | 5.99 | 2.47 |
|  | 11 | 0.48 | 1.50 | 1.65 | 3.66 | 0.54 | 0.40 | 0.22 |  | 2.74 |
|  | 12 | 0.81 | 2.23 | 2.36 | 3.92 | 1.31 | 0.56 | 3.32 | 6.92 |  |
| W2 |  | 50.73 | 42.90 | 50.96 | 40.54 | 48.10 | 46.62 | 43.64 | 38.60 | 38.92 |
| W3 | 2 |  | 3.04 | 5.97 | 7.60 | 6.10 | **9.12** | 7.84 | 6.71 | 4.33 |
|  | 3 | 3.04 |  | 5.64 | 6.20 | 6.45 | 6.11 | 5.81 | 5.57 | 4.09 |
|  | 5 | 5.97 | 5.64 |  | 6.04 | **8.52** | **8.55** | 6.57 | 4.87 | 4.79 |
|  | 6 | 7.60 | 6.20 | 6.04 |  | 3.31 | 3.73 | 3.43 | 4.46 | 5.76 |
|  | 7 | 6.10 | 6.45 | **8.52** | 3.31 |  | 4.86 | 6.91 | 5.64 | 6.30 |
|  | 8 | **9.12** | 6.11 | **8.55** | 3.74 | 4.86 |  | 4.11 | 4.44 | 5.70 |
|  | 9 | 7.84 | 5.81 | 6.57 | 3.43 | 6.91 | 4.11 |  | 3.97 | 5.01 |
|  | 11 | 6.71 | 5.57 | 4.87 | 4.46 | 5.64 | 4.44 | 3.97 |  | 2.94 |
|  | 12 | 4.33 | 4.09 | 4.79 | 5.76 | 6.30 | 5.70 | 5.01 | 2.94 |  |

Note. Bold indicates significant local dependence.

**Table S2. Local independence analysis of the 8-item subjective incompetence scale.**

| Index | Item | Item | | | | | | | |
| --- | --- | --- | --- | --- | --- | --- | --- | --- | --- |
|  |  | 2 | 3 | 5 | 6 | 7 | 9 | 11 | 12 |
| W1 | 2 |  | 5.71 | 3.74 | 3.60 | 3.15 | 2.19 | 4.00 | 3.29 |
|  | 3 | 0.74 |  | 2.92 | 4.38 | 0.39 | 2.44 | 4.09 | 1.86 |
|  | 5 | 1.42 | 2.48 |  | 3.33 | 1.04 | 2.01 | 3.89 | 1.95 |
|  | 6 | 0.48 | 1.90 | 1.26 |  | 1.00 | 0.43 | 1.52 | 1.42 |
|  | 7 | 1.64 | 2.89 | 2.02 | 4.81 |  | 2.86 | 4.68 | 2.47 |
|  | 9 | 1.01 | 2.85 | 1.77 | 4.51 | 1.36 |  | 5.33 | 2.39 |
|  | 11 | 0.46 | 1.48 | 1.59 | 3.24 | 0.49 | 0.19 |  | 2.51 |
|  | 12 | 0.45 | 1.88 | 1.81 | 2.87 | 0.94 | 2.40 | 5.26 |  |
| W2 |  | 35.11 | 32.98 | 38.65 | 31.28 | 34.97 | 33.61 | 31.58 | 27.44 |
| W3 | 2 |  | 2.52 | 5.06 | 5.69 | 5.44 | 6.55 | 6.60 | 3.26 |
|  | 3 | 2.52 |  | 5.12 | 4.91 | 4.77 | 4.69 | 6.14 | 4.84 |
|  | 5 | 5.06 | 5.12 |  | 5.84 | 7.32 | 6.17 | 5.06 | 4.08 |
|  | 6 | 5.69 | 4.91 | 5.84 |  | 2.94 | 3.51 | 3.40 | 5.00 |
|  | 7 | 5.44 | 4.77 | 7.32 | 2.94 |  | 5.59 | 4.47 | 4.44 |
|  | 9 | 6.55 | 4.69 | 6.17 | 3.51 | 5.59 |  | 3.60 | 3.50 |
|  | 11 | 6.60 | 6.14 | 5.06 | 3.40 | 4.47 | 3.60 |  | 2.31 |
|  | 12 | 3.26 | 4.84 | 4.08 | 5.00 | 4.44 | 3.50 | 2.31 |  |

Note. Bold indicates significant local dependence.

**Table S3. Scaled fit indices for the one-factor model of the SIS-8 according to confirmatory factor analysis.**

| Model | X^2^/df | CFI | TLI | RMSEA | SRMR |
| --- | --- | --- | --- | --- | --- |
| One-factor model | 46.819/20 | 0.929 | 0.901 | 0.076 | 0.045 |

Note. **CFI, Comparative Fit Index. TLI, Tucker-Lewis Index. RMSEA, Root Mean Square Error of Approximation. SRMR, Standardized Root Mean Square Residual.**
